# *FHIT*基因启动子甲基化与肺癌相关性的*meta*分析

**DOI:** 10.3779/j.issn.1009-3419.2014.03.09

**Published:** 2014-03-20

**Authors:** 义长 孙

**Affiliations:** 473058 南阳，南阳医学高等专科学校第一附属医院肿瘤内科 Department of Medical Oncology, the First Affiliated Hospital, Nanyang Medical College, Nanyang 473058, China

**Keywords:** 肺肿瘤, MGMT基因, 甲基化, *Meta*分析, Lung neoplasms, *FHIT* gene, Methylation, *Meta*-analysis

## Abstract

**背景与目的:**

抑癌基因启动子序列DNA甲基化与该基因在肿瘤细胞中表达下调有关。*FHIT*（fragile histindine triad）基因被认为是重要的肿瘤抑制基因，文献报道其启动子序列DNA在肿瘤组织中常发生甲基化改变。本研究采用*meta*分析的方法探讨*FHIT*基因启动子序列DNA甲基化与肺癌的相关性。

**方法:**

检索Pubmed、CNKI及万方数据库，收集公开发表的关于*FHIT*基因启动子序列DNA甲基化与肺癌相关性的论文。对比分析肺癌组织与其配对的癌旁/正常肺组织、支气管盥洗液或血浆中*FHIT*基因启动子序列DNA甲基化发生率有无差别。以优势比（odd ratio, OR）为效应量，采用STATA 11.0统计软件进行合并分析。

**结果:**

最终纳入符合要求的文献11篇，肺癌组织、癌旁/正常肺组织、支气管盥洗液和血浆中*FHIT*基因启动子序列甲基化率分别为*P*_median_=40.0%（0-68.3%）、*P*_median_=8.7%（0-35.0%）、*P*_median_=33.3%（17.1%-38.3%）和*P*_median_=35.9%（31.1%-50.8%）。肺癌中*FHIT*基因启动子序列甲基化率明显高于正常肺组织（OR=5.82, 95%CI: 3.74-9.06, *P* < 0.05），而与支气管盥洗液和血浆比较差别无统计学意义，（OR=1.55, 95%CI: 0.89-2.70, *P* > 0.05; OR=1.41, 95%CI: 0.90-2.20, *P* > 0.05）。

**结论:**

与癌旁/正常肺组织相比患者肺癌组织中*FHIT*基因启动子序列DNA甲基化率明显增高，该基因的启动子甲基化与肺癌的发生可能存在相关性。

## Abstract (en)

**Background and objective:**

Tumor-suppressor gene promoter DNA methylation in tumor cells is associated with its reduced expression. *FHIT* (fragile histindine triad) was one of the important tumor suppressor genes which was found hypermethylated in the promoter region in most of tumors. The aim of this study is to evaluate the relationship between *FIHT* gene promother methylation and lung cancer risk by *meta*-analysis.

**Methods:**

By searching Pubmed, CNKI and Wanfang, the open published articles related to *FHIT* gene promoter methylation and lung carcinoma risk were collected. The odds ratio (OR) and range of *FHIT* gene of cancer tissue of lung cancer patients compared with normal lung tissue, plasma and the bronchial lavage fluid were pooled by statistical software Stata 11.0.

**Results:**

Eleven studies were finally included in this meta-analysis. The median methylation rate were *P*_median_=40.0% (0-68.3%), *P*_median_=8.7% (0-35.0%), *P*_median_=33.3% (17.1%-38.3%) and *P*_median_=35.9% (31.1%-50.8%) in cancer tissue, NLT, BALF and plasm respectively. The pooled results showed the methylation rate in tumor tissue was much higer than that of NLT OR=5.82 (95%CI: 3.74-9.06, *P* < 0.05), but without statistical significance for BALF OR=1.55 (95%CI: 0.89-2.70, *P* > 0.05) and plasma OR=1.41 (95%CI: 0.90-2.20, *P* > 0.05).

**Conclusion:**

Hypermethylation of *FHIT* gene promoter region was found more frequent in cancer tissue than that of NLT which may demonstrated association between lung cancer risk and *FHIT* gene promoter methylation.

1966年Ohta及其同事^[1]^率先采用外显子捕获法在3号染色体短臂14带2区发现*FHIT*基因，其大小约1.5 MB，内部包括多个断裂位点。该基因编码一个由147个氨基酸残基组成的16.8 kDa的蛋白质，该蛋白被认为是HIT蛋白家族成员之一，其功能可能与Ap4A水解酶的功能相似。近年来的研究显示，*FHIT*基因启动子序列甲基化是导致其表达下调的重要表观遗传学调控机制之一，启动子序列甲基化在众多癌组织中被发现。肺癌组织中*FHIT*基因启动子序列DNA常呈现高甲基化状态，可能与肺癌的发生有关。本研究采用*meta*分析的方法对*FHIT*基因启动子甲基化与肺癌的相关性进行探讨。

## 1 文献与方法

### 1.1 文献检索

检索Pubmed、CNKI及万方等数据库，收集公开发表的关于*FHIT*基因启动子甲基化与肺癌相关性的研究。检索语种为英语和汉语，分别以“FHIT/ fragile histindine triad”、“lung cancer”、“lung carcinoma”、“methylation”为主题词和自由词，检索Medline、数据库；以“肺癌”“非小细胞肺癌”“肺肿瘤”“*FHIT*”基因，“甲基化”为关键词或题名检索CNKI和万方等中文数据库。

### 1.2 数据提取

采用双人平行摘录方法，由两位研究者独立阅读所获文献题目和摘要，在排除明显不符合纳入标准的试验后，对可能符合纳入标准的试验阅读全文以最终确定是否符合纳入标准。两位评价者交叉核对纳入试验的结果，对有分歧而难以确定是否纳入的试验通过讨论或由第三位评价者决定其是否纳入。提取内容包括：①一般资料：题目、作者姓名、发表日期、文献来源；②研究特征：研究对象的一般情况、各组患者的基线可比性、干预措施；③结局指标：每项研究的癌组织、正常肺组织/癌旁组织、支气管盥洗液和血浆中*FHIT*基因启动子序列DNA甲基化发生例数或发生率。

### 1.3 统计分析

阅读文献、整理数据、建立数据库并核校数据。采用癌组织与正常肺组织/癌旁组织、支气管盥洗液和血浆中*FHIT*基因启动子序列甲基化发生率的优势比OR为效应指标，并以95%可信区间（95%CI）表示，双侧*P* < 0.05认为有统计学意义。统计学异质性采用Q统计量的*I*^2^检验来分析，双侧*P* > 0.05认为各研究间不存在明显的异质性，采用固定效应模型（fixed effect model）合并数据；如果各研究间存在明显的异质性（*P* < 0.05），分析其异质性的来源，对可能导致异质性的因素进行亚组分析。若各研究组之间存在统计学异质性而无临床或方法学异质性或差异无统计学意义时，采用随机效应模型（random effect model）合并数据。如果各研究间异质性过大则不适合定量合成数据转而采用描述性分析。采用*Begg’s*法对发表偏倚进行量化检测。统计应用Stata 11.0统计软件完成。

## 2 结果

### 2.1 检索结果

最终纳入符合要求的文献11篇，来自中文数据库的文献9篇，英文数据库文献2篇，所有纳入的原始研究均提供了较为完整的数据资料（表 1）。

**1:** 本文纳入研究的基本特征 The general characteristics of included studies

Author	Number of cases		Control type	Methods	Region	Year
	Tumor	Control				
Zochbauer-Muller^[2]^	107	104	NLT	qMSP	USA	2001
Zochbauer-Muller^[2]^	107	35	BALF	qMSP	USA	2001
Hsu^[3]^	63	63	Plasm	MSP	USA	2007
Yang^[4]^	53	57	NLT	MSP	China	2007
Yu^[5]^	45	45	BALF	MSP	China	2007
Yu^[5]^	45	45	Plasm	MSP	China	2007
Yang^[6]^	60	60	NLT	MSP	China	2007
Yu^[7]^	45	23	NLT	MSP	China	2007
Kang^[8]^	60	15	NLT	MSP	China	2009
Li^[9]^	52	52	NLT	MSP	China	2009
Song^[10]^	78	78	NLT	MSP	China	2011
Kang^[11]^	47	47	BALF	MSP	China	2011
Kang^[12]^	53	53	Plasm	MSP	China	2011
BALF: bronchoalveolar lavage fluid; NLT: normal lung tissue.						

### 2.2 不同组织甲基化率比较

肺癌组织、癌旁/正常肺组织、支气管盥洗液和血浆中*FHIT*基因启动子序列甲基化率分别为*P*_median_=40.0%（0-68.3%）、*P*_median_=8.7%（0-35.0%）、*P*_median_=33.3%（17.1%-38.3%）和*P*_median_=35.9%（31.1%-50.8%）。

### 2.3 *FHIT*基因启动子甲基化与肺癌相关性

肺癌中*FHIT*基因启动子序列甲基化率明显高于正常非组织（OR=5.82, 95%CI: 3.74-9.06, *P* < 0.05），而与支气管盥洗液和血浆比较差别无统计学意义（OR=1.55, 95%CI: 0.89-2.70, *P* > 0.05;OR=1.41, 95%CI: 0.90-2.20, *P* > 0.05）（图 1，图 2）。

**1:**
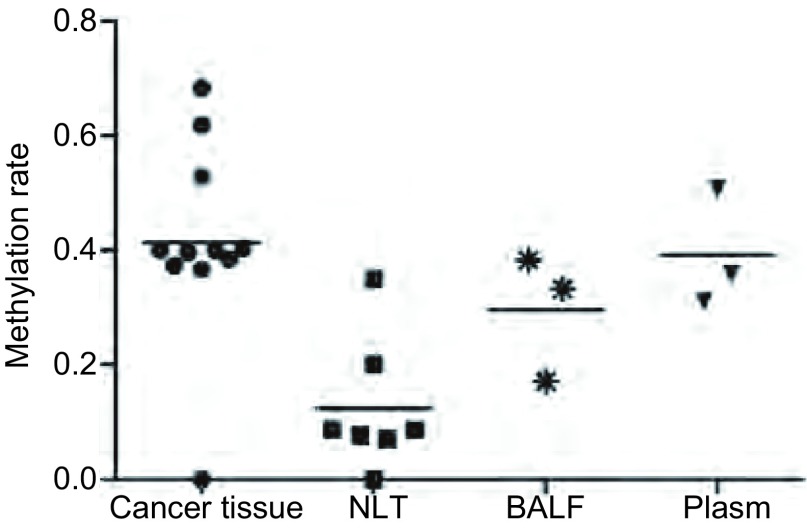
不同组织中*FHIT*基因启动子序列甲基化发生率 Hypermethylation frequency of *FHIT* gene in different tissue types

**2:**
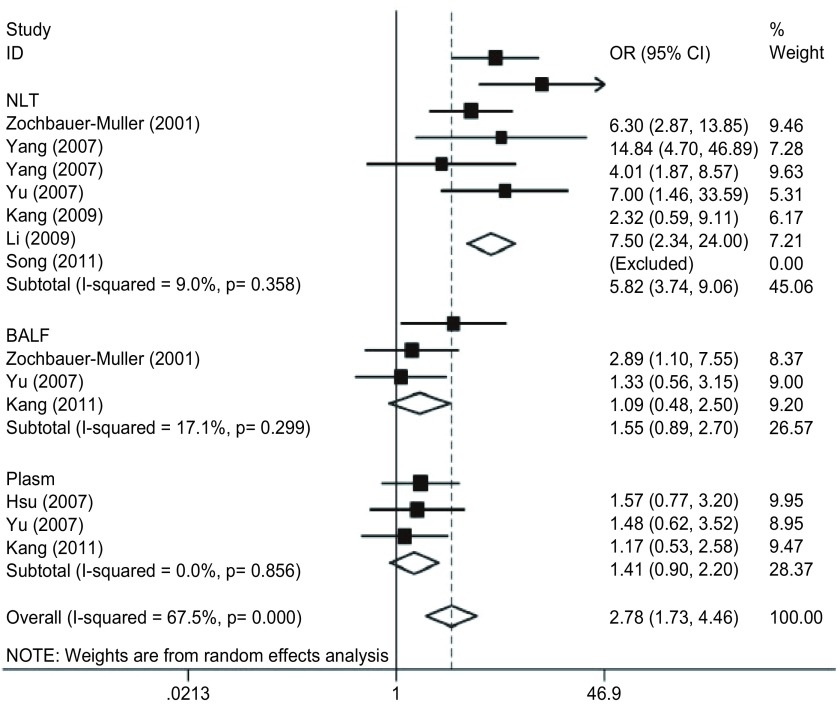
*FHIT*基因启动子甲基化与非小细胞肺癌关系的森林图 Forest plot of *FHIT* gene promother methylation in cancer tissue compared with controls

### 2.4 发表偏倚

*Begg’s*法量化评价发表偏倚，*Begg’s*检验中Pr > |z|=0.170，*P* > 0.05，图中各点沿中间水平线均匀分布，基本位于预计95%CI内（图 3），结果提示不存在明显的发表偏倚。

**3:**
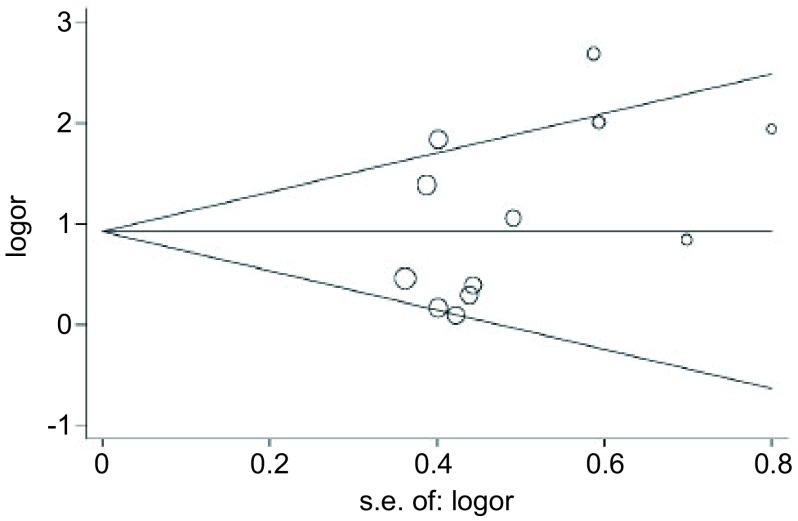
*Begg's*法评价发表偏倚 Publication bias evaluation by *Begg's*

## 3 讨论

肺癌是目前发病率最高的恶性肿瘤，其死亡率在男性为第一位，女性为第二位。根据新近的流行病学资料^[13]^显示，每年全球肺癌新发病例高达120万人，死亡约为100万人。肺癌已成为威胁人类健康的重要原因之一。吸烟是肺癌的主要危险因素，流行病学资料显示，90%的肺癌与吸烟有关^[14]^。此外，全基因组范围内的抑癌基因失活或表达下调可能是肺癌发生的分子生物学机制之一。DNA甲基化是表观遗传学调控基因表达的重要机制之一，大量研究^[15, 16]^显示，抑癌基因启动子序列CpG岛区域存在DNA高甲基化状态，启动子甲基化可进一步抑制该基因的转录，从而导致其蛋白表达量下调。肺癌组织中发现存在多种抑癌基因启动区域的高甲基化状态，如*P16ink4a*、*MGMT*、*APC*及*FHIT*等。*FHIT*基因编码一个由147个氨基酸残基组成的16.8 kDa的蛋白质，该蛋白被认为是HIT蛋白家族成员之一，其功能可能与Ap4A水解酶的功能相似。有研究^[2, 3]^显示，FHIT基因启动子序列甲基化发生率在肺癌组织中明显高于癌旁组织/正常肺组织，但各研究存在一定的差异，且由于样本量较小统计效能较低，因此，*FHIT*基因启动子序列DNA高甲基化与肺癌发生关系值得进一步研究。

本研究采用循证医学的方法探讨*FHIT*基因启动子序列DNA甲基化与肺癌的相关性。最终检索并纳入关于*FHIT*基因启动子序列甲基化与肺癌关系的研究11项，*meta*分析结果显示，11篇文献中均提供了各项组织标本中*FHIT*基因启动子序列甲基化发生率，肺癌组织、癌旁/正常肺组织、支气管盥洗液和血浆中*FHIT*基因启动子序列甲基化率分别为*P*_median_=40.0%（0-68.3%）、*P*_median_=8.7%（0-35.0%）、*P*_median_=33.3%（17.1%-38.3%）和*P*_median_=35.9%（31.1%-50.8%）。肺癌中*FHIT*基因启动子序列甲基化率明显高于正常肺组织（OR=5.82, 95%CI: 3.74-9.06, *P* < 0.05），而与支气管盥洗液和血浆比较差异无统计学意义（OR=1.55, 95%CI: 0.89-2.70, *P* > 0.05;OR=1.41, 95%CI: 0.90-2.20, *P* > 0.05）。结果提示，与癌旁/正常肺组织相比患者肺癌组织中FHIT基因启动子序列DNA甲基化率明显增高，该基因的启动子甲基化与肺癌的发生可能存在相关性。

本研究从分子流行病学角度提供了*FHIT*基因启动子甲基化与肺癌发生存在相关性的证据，但关于*FHIT*基因启动子甲基化抑或表达下调与肺癌发生的分子生物学机制目前仍不十分明了。同时，*FHIT*基因启动子甲基化只是其表达调控的重要途径之一，且大多数情况下启动子序列DNA甲基化只在mRNA转录水平上导致其表达下降，而对于转录后修饰及mRNA翻译及降解层面并不起作用。因此，*FHIT*基因启动子序列DNA甲基化与肺癌发生相关性的分子学机制有待进一步研究。

在本研究中，*FHIT*基因启动子甲基化与肺癌的发生有关，该基因启动子甲基化在肺癌的发生过程中可能起重要作用，同时也提示甲基化转移酶抑制剂可能给肺癌治疗方面提供重要的契机。
